# A Novel Genotype of GB Virus C: Its Identification and Predominance among Injecting Drug Users in Yunnan, China

**DOI:** 10.1371/journal.pone.0021151

**Published:** 2011-10-06

**Authors:** Yue Feng, Wenhua Zhao, Yuemei Feng, Jiejie Dai, Zheng Li, Xiaoyan Zhang, Li Liu, Jie Bai, Huatang Zhang, Ling Lu, Xueshan Xia

**Affiliations:** 1 Faculty of Environmental Science and Engineering and Faculty of Life Science and Technology, Kunming University of Science and Technology, Kunming, Yunnan, China; 2 The Key Laboratory of Tropical and Subtropical Animal Viral Diseases in Yunnan province, Kunming, Yunnan, China; 3 Key Laboratory of Animal Models and Human Disease Mechanisms of Chinese Academy of Sciences and Yunnan Province, Kunming Institute of Zoology, Kunming, China; 4 Institute of Medical Biology, Chinese Academy of Medical Science and Peking Union Medical College, Kunming, Yunnan, China; 5 The Clinical Laboratory Center of Yunnan Province, Affiliated Kunhua Hospital of Kunming Medical College, Kunming, China; 6 Research Center of Shanghai Public Health Clinical Center, Institutes of Biomedical Sciences, Fudan University , Shanghai, China; 7 The Viral Oncology Center, Department of Pathology, University of Kansas Medical Center, Kansas City, Kansas, United States of America; 8 Laboratory of Hepatology, 3rd Affiliated Hospital, Sun Yat-sen University, Guangzhou, Guangdong, China; Centers for Disease Control and Prevention, United States of America

## Abstract

GB virus C (GBV-C) is prevalent globally and particularly among individuals at risk of parental exposures. Based on genetic diversity, this virus is now classified into six genotypes and many subtypes with distinct geographical distribution. In this study, 120 Injecting Drug Users (IDUs) were recruited from Yunnan province, China. Among them, 43 (35.8%) were positive for GBV-C RNA, 70 (58.3%) and 103 (85.8%) sero-positive for HIV-1 and HCV respectively. This revealed 18.3% of IDUs having GBV-C/HIV/HCV triple infection, which is significantly higher than 7.5% of GBV-C/HIV-1 and 10% of GBV-C/HCV dual infection rates (P<0.05). Based on 5′UTR sequences, the identified 43 viral isolates can be classified into three phylogenetic groups: one (2.3%) and two (4.7%) belonged to genotype 3 and 4, respectively, and the remaining 40 (93%) formed a new group with 97% of bootstrap support. This new GBV-C group was further confirmed by characterizing the E2 region and full-length genome sequences. Analysis of 187 nt 5′UTR sequence showed three previous reported isolates from Southeast Asia were re-classified into this new group. It implies they have the same origin with strains from Yunnan. Although we provisionally assigned this new group as GBV-C genotype 7, a simpler five groups of GBV-C nomenclature is recommended. Genotype 4, 6 and the newly designated genotype 7 could be reclassified as one group, which may represent a single GBV-C genotype. The classification of the other four groups was corresponding to that of previous reported genotype 1, 2, 3 and 5. Furthermore, the diversity of amino acid sequence in the E2 region was analyzed. The inhibitory effect of GBV-C genotype 7 on HIV-1 cell entry could be deduced. Since GBV-C may have a beneficial effect on AIDS disease progression and interact with HCV during co-infection, this finding may raise interests in future studies on this virus that was previously thought to be a “non-pathogenic virus”.

## Introduction

Discovered by two independent groups in the mid-1990s, GB Virus C (GBV-C)/Hepatitis G Virus (HGV) has now been classified as a member of the *Flaviviridae* family. GBV-C has a single-stranded positive RNA genome of about 9.3 kb and contains a single open reading frame (ORF) encoding two structural (E1 and E2) and five non-structural (NS2, NS3, NS4, NS5A, and NS5B) proteins [1], [2]. Although with 25–30% of amino acid sequence similarity to hepatitis C virus (HCV), the GBV-C genome lacks the core region and hypervariable region of E2 gene. In addition, its 3′-untranslated region (UTR) displays a less complex organization [3].

GBV-C infection has been found worldwide. High prevalence is observed among subjects with the risk of parenteral exposures. The subjects include those with exposure to blood and blood products, those on maintaining hemodialysis, and those with intravenous drug using [4]. Sexual contact and vertical route may also mediate GBV-C transmission [5]. Due to shared transmission modes, co-infection with GBV-C is common among people infected with HIV-1 and/or HCV. Approximately, 10%–25% of chronic hepatitis C patients and 14%–36% of Injecting Drug Users (IDUs) seropositive for HIV-1 show the evidence of GBV-C co-infection [6], [7]. The much higher GBV-C triple infection rate of 30%–36% among individuals with HIV/HCV co-infection has been reported by recent investigations [8], [9].

Comparing with HCV, the genetic diversity of GBV-C is lower with 11–14% of nucleotide difference in the polyprotein coding region between genotypes [10]. Currently, GBV-C has been classified into six genotypes and many subtypes based on their sequence diversity of either full genome length or a particular genomic range. Geographically, these genotypes and subtypes showed distinct distribution patterns. In general, genotype 1 is predominant in African and is divided into five subtypes [11]. Genotype 2 has three subtypes [12] and is found in Europe and America. Genotype 3 is the most common in Asia including Japan and China. In contrast, genotype 4 is predominant in Southeast Asia, and genotype 5 is only seen in South Africa. Recently, genotype 6 is proposed with its sequences discovered in Indonesia [10].

Although GBV-C infection has not been found in association with any particular disease, its co-infection with HIV-1 may produce some favorable outcomes, with a lower mortality rate, slower disease progression, and longer survival term [13], [14]. In addition, GBV-C genotypes 2 and 5 have been found in connection with a more delayed AIDS progression [10], [15], [16]. Recent studies further show that HIV-1 infection and its downstream viral replication can be inhibited by GBV-C E2 fusion peptide [17]. The mutation of a functional fragment (269–286 GTEVSEALGGAGLTGGFY), which specifically binds to HIV-1 gp41, may impede this action [18]. Collectively, these findings suggest that GBV-C genetic diversity has impacts on HIV-1 replication and therefore, there is a need to study its genetic diversity in the context of co-infection with other blood-borne viruses.

The Yunnan province is situated in southwest China. It borders Southeast Asia countries of Laos, Vietnam and Myanmar to the south. As a hub on the trans-continental drug trafficking road, Yunnan has played a critical role in spreading many blood-borne infections in China [19]. For example, HIV-1 is introduced from the Golden Triangle to Yunnan and then spread to other provinces of the country [20]. It has been considered that the recombinantion of current predominant HIV-1 CRF_07 &_08 in China occurrs in Yunnan, as the origin of their national wide prevalence [21], [22]. For HCV, the unique HCV genotypes distribution and existence of two novel subtypes have been also revealed among this population [23], [24]. Moreover, in this region, the general population has been demonstrated to be threated with the more frequent HIV-1 spreading from IDUs through sex transmission [25].The high infection rates of HIV-1 and HCV among IDUs have been reported [26], [27]. Furthermore, the change of HIV-1 and HCV epidemic in neighboring regions, such as Guangxi and Sichuan provinces was found to be on-going under the impact of their prevalence in Yunnan [28], [29].

Some studies have revealed that GBV-C infection occurs among IDUs in Southern China [30]. However, there are no reports on the GBV-C genotypes distribution among IDUs with co-infection with HIV-1and/or HCV. Thus, the major purpose of this study was to determine the prevalence of GBV-C genotypes among IDUs. In this report, GBV-C infection was tested among 120 IDUs who were recruited from five prefectures of Yunnan province. Dual or triple infection with HIV-1 and HCV were also studied. After characterization of 5′UTR and nearly entire E2 region sequences, a novel GBV-C genotype was discovered and its predominance among the IDUs was revealed. With sequencing and subsequent analysis of three full-length genomes, this novel GBV-C genotype was confirmed. Furthermore, the analysis on E2 amino acid sequences of GBV-C was performed to evaluate its possible effect on co-infected HIV-1.

## Results

### GBV-C detection and its co-infection with HIV-1 and HCV

**Figure 1:**
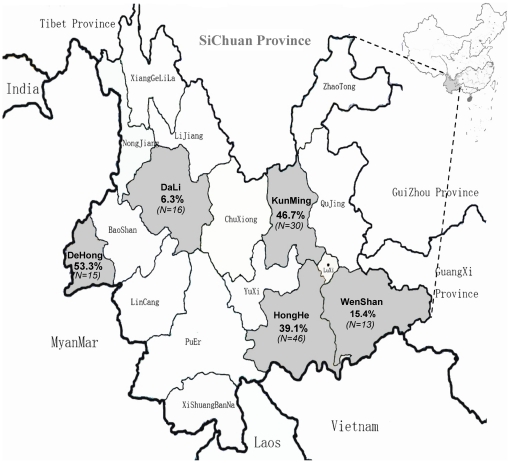
Map of the Yunnan Province in the People's Republic of China. Gray areas mark the five prefectures the 120 IDUs were recruited. GBV-C positive rates were shown in percentages.

A total of 120 IDUs were recruited from six drug detoxification centers in five prefectures of Yunnan province, China. Among these prefectures three (Dehong, Honghe, Wenshan) border Myanmar and Vietnam, while two (Kunming and Dali) are located at the central part of the province (Figure 1). Of the 120 IDUs, 102 were males and 18 females. Their ages ranged between 20 and 65 years old, with an average of 33 years old (SD = 6.78). After amplification of 5′UTR (378bp) using nested RT-PCR, GBV-C RNA was detected in 43 IDUs (35.8%). In the sampled prefectures, the GBV-C RNA positive rates among IDUs were 46.7% for Kunming, 39.1% for Honghe and 53.3% for Dehong. There were no significant differences between these prefectures. However, their GBV-C RNA positive rates were significant higher than those for Dali (6.3%) and Wenshan (15.4%). Detection of GBV-C anti-E2 revealed a GBV-C seropositive rate of 25.83% (31 IDUs). Only two cases with clinically confirmed AIDS were positive for both GBV-C anti-E2 and GBV-C RNA. Furthermore, 70 (58.3%) and 103 (85.8%) of all the recruited IDUs were detected to be sero-positive for HIV-1 and HCV, respectively. Of them, 61 were sero-positive for both HIV-1 and HCV (Table S1). These mono or double infection rates of HIV-1 and HCV were consistent with the previous reports [27]. Among the 43 IDUs positive for GBV-C RNA, nine (7.5%) were co-infected with HIV-1, 12 (10%) were co-infected with HCV, and 22 (18.3%) co-infected with both HIV-1 and HCV (Figure S1). Chi-square test showed that the rate of GBV-C/HIV-1/HCV triple infection was significantly higher than those of GBV-C/HIV-1 and GBV-C/HCV dual-infections (*P*<0.05).

### GBV-C genotypes distribution and genotype 7 identification

**Figure 2:**
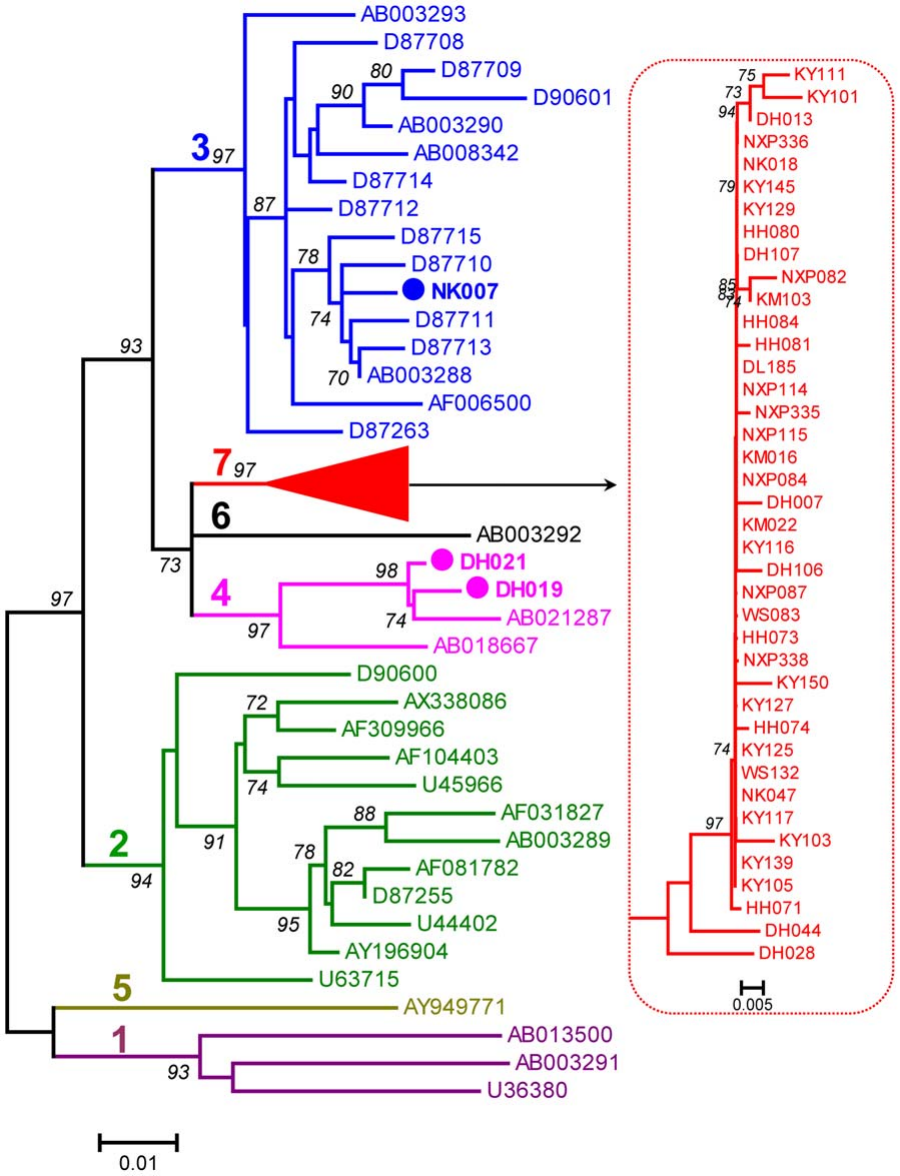
Phylogenetic tree based on 5′UTR sequences of 378 nt in length. The sequences corresponding to nucleotide positions -210 to 148 in the genome of prototype GBV-C isolate U36380. The known six GBV-C genotypes are indicated with number 1–6. Reference GBV-C sequences is indicated with isolate names followed by their Genbank accession numbers. The neighbor-joining tree was constructed using the Jukes-Cantor distances. Bootstrap values are shown in bold.

**Figure 3:**
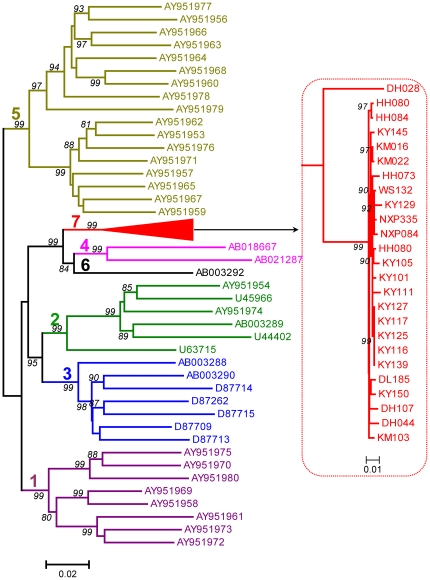
Phylogenetic tree reconstructed with GBV-C E2 region sequences. These sequences were each 1004 bp in length, corresponding to nt 610–1613 in the U36380 genome. Otherwise all the labels are as the same as shown in figure 2.

**Figure 4:**
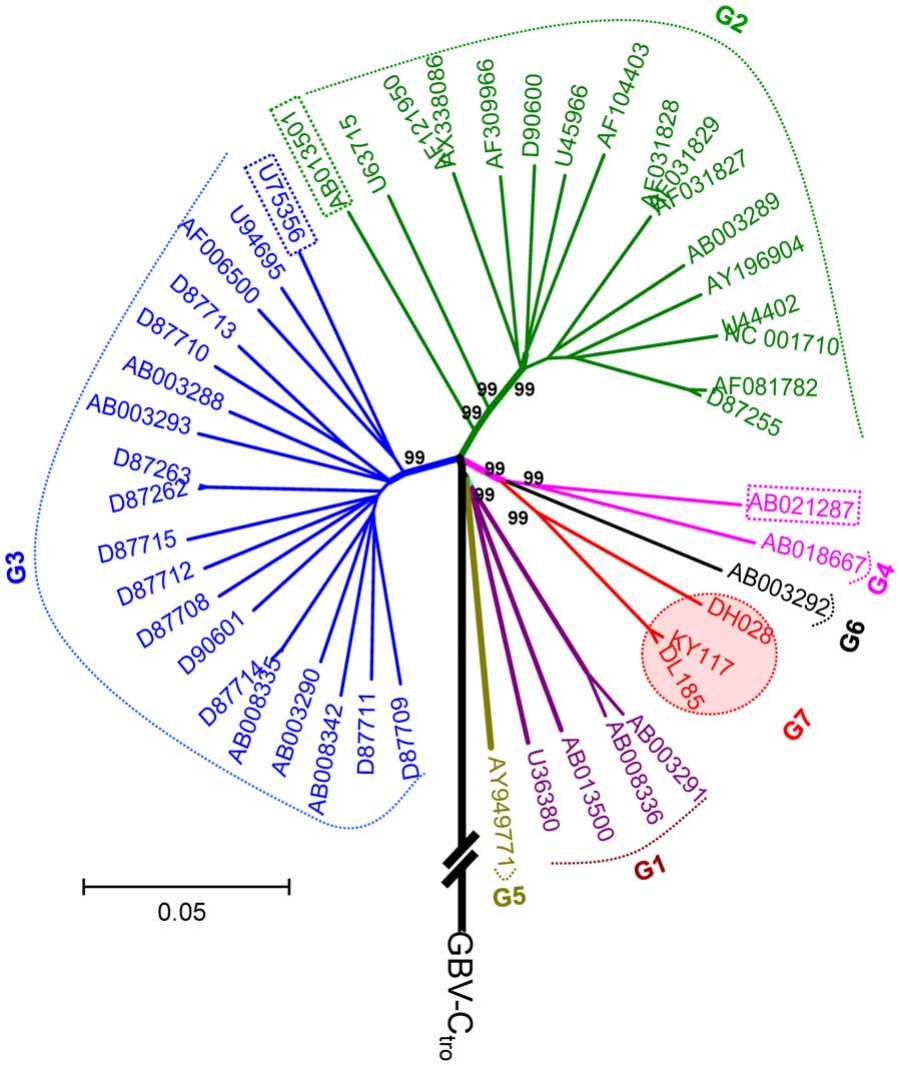
Rooted phylogenetic tree based on the full-length GBV-C genome sequences of 48 isolates. The isolate GBV-C tro (Genbank accession no. AF070476) was used as an outgroup. The symbol//indicates a trimmed branch. Putative recombinant isolates are boxed with dashed lines. Otherwise all the labels are as the same as shown in figure 2.

The amplification of GBV-C 5′UTR was successful for 43 IDUs and the expected amplicons corresponded to nucleotides -414 to -37 of the prototype GBV-C genome (U36380). Based on the 5′UTR sequences, a phylogenetic tree was reconstructed. The tree showed that the obtained 5′UTR sequences were divided into three phylogenetic groups. A sequence (2.3%) from Kunming (KM07) was classified into genotype 3, the reported predominant genotype in China. Two sequences (4.7%) from Dehong (DH019 and DH021) were classified into genotype 4, which was common in Southeast Asia. Unexpectedly, the remaining 40 sequences (93%) formed a solid cluster with a 97% of bootstrap support. This cluster was distinct from the known six GBV-C genotypes, suggesting the existence of a new genotype (Figure 2). For a better illustration, the nearly entire E2 region sequence of GBV-C was amplified from 25 IDUs. The amplicons were 1004 nt in length and corresponded to nt 610-1613 of the U36380 genome. The E2 region's amplification of remaining 18 samples was failed, probably due to low viral load, which is difficult to amplify the long RNA fragment. Phylogenetic tree based on these E2 region sequences showed grouping result consistent with that analyzed with GBV-C 5′UTR. These sequences were tightly clustered into a novel group that has a bootstrap support of 99% (Figure 3). For further verification, three full-length GBV-C genomes (KY117, DL185, and DH028) were characterized. This was done using the strategy shown in Figure S2 with each genome amplified by 13 overlapping fragments. To identify the position of the root of a phylogenetic tree, the nucleotide sequence of the GBV-C chimpanzee variant, GBV-C_tro_ was included as the out-group. In this rooted phylogenetic tree (Figure. 4), the three full-length GBV-C sequences formed a solid cluster. This cluster was distinct from the known six GBV-C genotypes and had a 99% of bootstrap support. Within this cluster, two small branches were contained, one leading to the KY117 and DL185 isolates and the other leading to DH028. As a whole, this cluster was linked by a long internal branch, which very similar to the forming of GBV-C genotypes 4 and 6. To exclude the potential recent events of viral recombination, similarity plotting was performed. Comparing the three full-length GBV-C genome sequences with a series of references, representing the known six GBV-C genotypes, no meaningful finding was obtained (figures not shown). These results may support a designation of the three full-length sequences as a new GBV-C genotype we temporarily assigned genotype 7. Based on the tree of partial and full-length genome sequences, genotype 4, 6 and the newly designated genotype 7 could be reclassified as one group, which may represent a single GBV-C genotype, as more GBV-C variants are characterized. The classification of other four groups was corresponding to that of previous reported genotype 1, 2, 3 and 5. The genotype 2/3 recombinant (AB013501) and genotype 1/2/3 recombinant (U75356) were belonged to genotype 2 and 3 groups respectively. It accounts to the five groups or genotypes could be suggested in the modified nomenclature system.

The three full-length GBV-C sequences, KY117, DL185, and DH028, were further characterized. They all had a 389 nt of 5′UTR, followed by a single ORF of 8529 nt and a 316 nt of 3′UTR. They shared common sizes in seven protein-encoding regions: E1(608 nt/212 aa), E2(1161 nt/387 aa), NS2(843 nt/281 aa), NS3(2031 nt/677 aa), NS4(945 nt/315 aa), NS5A(1245 nt/415 aa), and NS5B(1692 nt/564 aa). Against six reference sequences representing six known GBV-C genotypes, nucleotide similarities were calculated (Table 1). Among the three genomes, KY117, DL185, and DH028, the nucleotide similarities ranged from 91.2 to 99.2% (mean 95.2%) over the complete genome length and from 91.0 to 99.2% (mean 95.1%) over the entire ORF range. Comparing KY117, DL185, and DH028 with the six references, the nucleotide similarities were of 86.2 to 89.0% (mean 87.6%) over the complete genome length and of 86.1 to 88.8% (mean 87.4%) over the entire ORF. Of the 7 protein-encoding regions, NS5A (90.2%) and NS5B (88.9%) were the most conserved, while E2 (mean 86.4%) and NS4 (mean 86.3%) the highest variable. Inter-genotype similarity of GBV-C genotype 4 (mean 88.9%) and 6 (mean 88.8%) were higher than those of genotype 1 (mean 86.3%) and 2 (mean 86.7%).

**Table 1:** Nucleotide similarities (%) of three genotype 7 isolations to 6 reference sequences in different genomic regions.

Compared a	Genome^b^	ORF	5′UTR	E1	E2	NS2	NS3	NS4	NS5A	NS5B	3′UTR
1(HGU36380)	86.5-86.9	86.4-86.8	87.2-87.7	85.4-87.0	84.9-86.3	84.2-85.3	85.1-85.4	85.8-86.1	89.2-89.8	88.3-88.6	95.9-96.8
2(HGU44402)	86.2-86.4	86.1-86.3	88.7-89.5	84.9-85.5	84.0-84.2	84.9-85.2	85.7-86.4	83.7-84.6	88.1-89.3	87.4-88.6	96.5-97.5
3/GT230(D90601)	87.6-88.3	87.5-88.2	89.5-90.0	86.8-87.7	85.6-87.0	87.7-88.5	86.1-86.7	87.2-88.8	89.4-89.7	89.3-90.2	96.5-97.5
4/MY14(AB018667)	88.8-89.0	88.6-88.8	91.8-93.6	87.7-89.3	88.1-88.7	87.0-87.4	86.7-87.6	87.9-88.7	92.0-92.3	88.7-90.3	96.2-97.1
5/D50(AY949771)	86.9-87.3	86.9-87.2	87.2-88.7	87.3-87.7	84.2-85.0	84.1-86.4	84.2-85.4	86.8-88.3	89.8-91.0	89.2-90.0	∼^c^
6/G05BD(AB003292)	88.6-88.8	88.5-88.7	90.0-91.6	88.5-91.0	88.5-88.8	89.1-89.3	85.7-86.2	88.5-88.8	90.8-90.9	89.0-89.8	∼^c^
KY117 vs DL185	99.2	99.2	98.9	99.8	99.2	99.2	99.5	98.6	99.2	99.1	99.7
KY117 vs DH028	91.2	91.1	94.9	92.9	91.5	90.7	89.9	89.0	92.9	91.5	98.7
DL185 vs DH028	91.2	91.0	95.4	93.1	91.1	90.3	89.7	89.1	92.9	91.8	99.0

a Isolates to which the three genotype 7 sequences were pairwise compared. Each genotype was represented by one isolate as indicated with its Genbank accession number shown in parenthesis.

b Over the entire genome length.

c D50 and G05BD lack the 3’UTR.

### Co-analysis of reference GBV-C 5′UTR sequences

**Figure 5:**
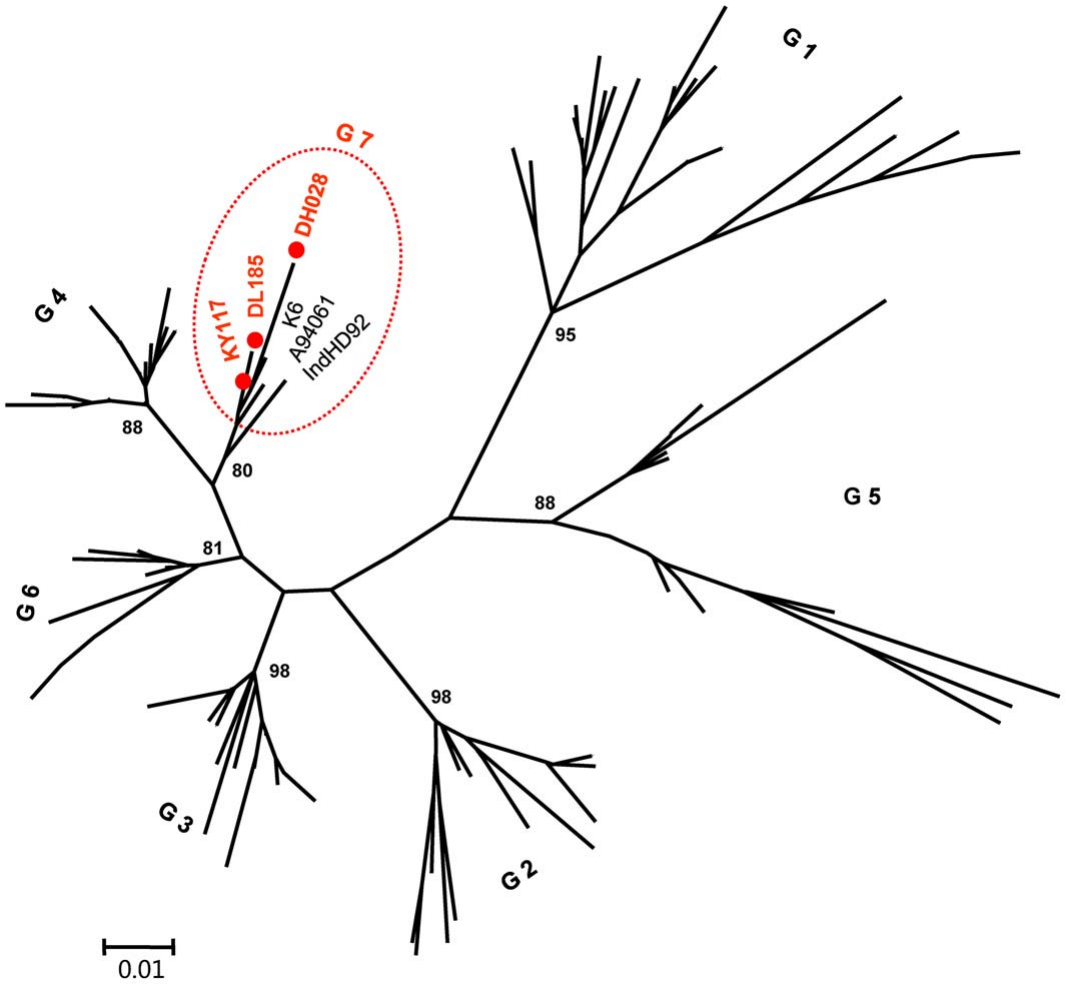
Phylogenetic tree based on partial 5′UTR region sequences. These sequences were each 187 nt in length, corresponding to nucleotides -398∼-202 in the genome of GBV-C isolate U36380. The tree was constructed using the Minimum Evolution method. Except for G7, all the isolates were adopted from the previous report by Muerhoff et al. [2006].

Among the detected 43 GBV-C isolates, genotype 7 was the most frequent accounting for 93%. In contrast, only one and two isolates belonged to genotype 3 and 4, respectively, accounting for 2.3% and 4.7%. This GBV-C genotypes distribution pattern is vastly different from those previously reported from China, in which genotype 3 was most predominant [31], [32]. This pattern looks also different from that in Southeast Asia where GBV-C genotypes 4 and 6 were common [10]. Regardless, the 43 GBV-C isolates all had an origin from Yunnan province proximity to Southeast Asia. To verify this incongruity, a further analysis was performed with 187 nt 5′UTR sequences, corresponding to nt -398 to -202 of the U36380 genome. Three references are included for this co-analysis. Among them A94061 and K6 (HGU91716 and HGU91721) were unassigned isolates from Thailand. IndHD92 was from a hepatitis patient in Indonesia, and it had been previously grouped into GBV-C genotype 4 [33]. With an 81% of bootstrap support, the three references were grouped with KY117, DL185, and DH028 that were completely sequenced in this study and assigned into genotype 7 (Figure 5). This analysis supplies a piece of evidence that GBV-C genotype 7 is also circulating in Southeast Asia.

### Analysis of amino acid sequences of E2 region

**Figure 6:**
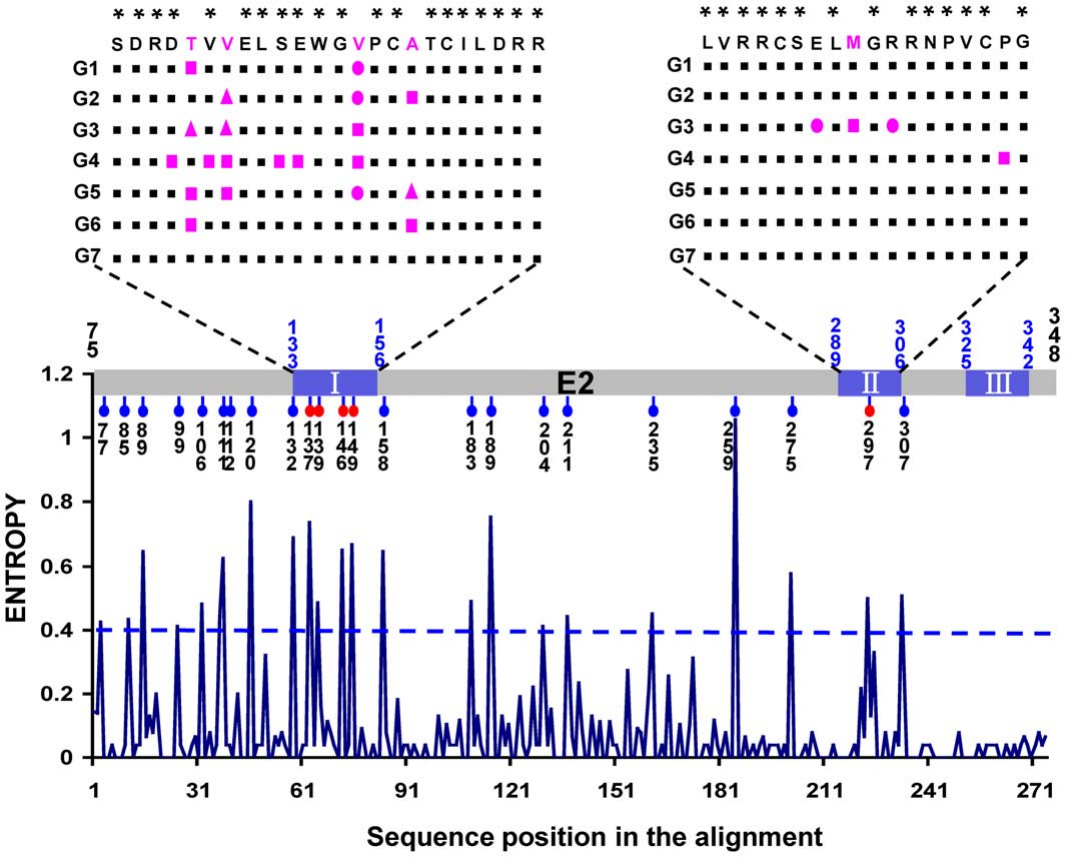
Analysis on amino acid sequences of E2 region. The entropy score (a mathematical measure of variability) at amino acid positions 75-348 within E2 region, analyzed with 158 GBV-C isolates. The gray bar indicates the amino acid positions starting from 75 and ending at 348. Within the gray bar a blue area indicates the three putative GBV-C E2 peptides inhibited HIV infection corresponding to aa 133–156, 289–306 and 325–342. Five red and 18 blue dots linking under the gray bar represent the 23 positions having the entropy score ≥0.4 (regarded as high variability) hat is measure by the scale on the left. A curve under the gray bar plots the entropy score against each amino acid position that is numbered by the ruler at the bottom. The blue area of the gray bar is further expanded; it shows an alignment of the consensus amino acid sequences from seven GBV-C genotypes spanning the blue area. In the alignment, the reported HIV-1 entry blocking peptide domain of GBV-C E2 region are cited as the consensus sequence. Symbol * indicates the site with variation lower than 10%, which considered as conservation site. For comparison, 21 reference strains of genotype 1, 64 strains of genotype 2, 26 strains of genotype 3, 2 strains of genotype 4, 18 strains of genotype 5, 2 strains of genotype 6 and 25 strains of our discoved genotype 7 were apopted. The pink little tri-angle, diamond and disk represent the mutation in this site account for 10%-20%, 20-50% and more than 50% of total cited strains in this genotype, respectively.

It has been confirmed that certain E2 domains of GBV-C may interfere with HIV-1 cellular entry by interaction with fusion peptide-Vesicle [17], [34], [35]. These domains are considered as the potential candidate for AIDS treatment and prevention. In this study, a mathematical measure of entropy was performed to evaluate the diversity of amino acids within a partial E2 region, corresponding to 75 to 348 of the GBV-C E2 poly-protein (Figure 6). This partial E2 region contains three relevant domains (E2_133–156_, E2_289–306_ and E2_325–342_), which have been proved to inhibit HIV entrance into target cell [36]. A total of 157 GBV-C sequences belonging to seven GBV-C genotypes respectively, were collected for analysis. Our data revealed 22 sites of analyzed sequences had significant mutation (the entropy scores >0.4). Among these sites, four mutation sites locate in the E2_133–156_ region, only one in E2_289–306_ region, and no one in E2_325–342_ region. The more mutation sites of E2_133–156_ may confer its impact on the interaction of GBV-C and HIV-1. In alignment, the consensus amino acid sequence (U45966) has been recently demonstrated to block HIV-1 entry by interference with gp41-mediated cell-cell fusion [36]. In these there investigated domains, the amino acid sequence of our discovered genotype 7 was totally consistent with that of the reported HIV-1 infection inhibitory peptide. The results suggest that the co-infection of GBV-C genotype 7 may depress the replication of HIV-1 and further delay AIDS disease progression.

## Discussion

In this study, the infection with HIV-1, HCV and GBV-C was investigated among 120 IDUs who were recruited in five prefectures of Yunnan province, China. We found the overall GBV-C RNA positive rate (43/120, 35.8%) was agreed with previous reports that GBV-C infection rate among IDUs ranged from 17.9% to 58.0% [30], [37], [38]. Among the total recruited subjects, 70 (58.3%) and 103 (85.8%) were found to be anti-HIV and anti-HCV positive, respectively. This accounted for 18.3% of IDUs having the GBV-C/HIV-1/HCV triple infection, 7.5% and 10.0% IDUs having the GBV-C/HIV-1 and GBV-C/HCV dual infection. Significantly, the rate of GBV-C/HIV-1/HCV triple infection was higher (P<0.05) than the rates of GBV-C/HIV-1 and GBV-C/HCV dual infection. Notably, no one was detected to be GBV-C mono-infection. It seems that the transmission of GBV-C though unsafe syringe using is not effective as HCV or HIV-1. In addition, a question is raised – if individuals with HIV-1/HCV dual infection are more susceptible to opportunistic infection such as with GBV-C [39]? Yet, an explicit answer to this question requires more analyses. Detection of GBV-C anti-E2 may be only indicative but not conclusive, because it indicates either a past or an active GBV-C infection depending on individuals. In this study, the majority of anti-E2 positive IDUs (29/31) were RNA negative, for whom past GBV-C infection was suggested [40]. However, in two IDUs who were clinically confirmed to have AIDS, both GBV-C anti-E2 and GBV-C RNA were positive, for whom active GBV-C infection was approved. Since these two patients were also positive for anti-HIV, other possibilities could exist. A simultaneous detection of the three markers, anti-HIV, GBV-C anti-E2, and GBV-C RNA, may reflect the fact that these individuals have more serious immunity damage; they may have produced higher titer of virus.

For GBV-C genotyping, different methods have been used. These included RT-PCR with specific primers, restriction fragment length polymorphism, and direct DNA sequencing [41]. Among these methods, DNA sequencing is the gold standard and the most preferred but requires phylogenetically informative regions to be analyzed. For such a purpose, the highly conserved 5′-UTR or its partial fragment is often concerned [10]. Some researchers have argued that phylogenetic analysis of this region may not always supply a solid genotyping result. In contrast, sequencing the complete or partial E2 region can provide stronger information, which is nearly in a complete accordance with sequencing the full-length GBV-C genomes [42]. Ultimately, defining a new viral variant or assigning a novel viral group constantly requires the full-length viral genome(s) being characterized. Based on this premise, a stepwise strategy was used in this study to process samples collected from 120 IDUs. Firstly, sequence of GBV-C 5′UTR was screened for all samples and this resulted in 43 samples (35.8%) positive. Of the obtained 43 isolates, 40 (93%) were classified into a new phylogenetic group. Secondly, the complete GBV-C E2 region was amplified in samples positive for GBV-C 5′UTR. This was done to verify the new GBV-C group. From 24 IDUs the E2 region sequences were amplifiable and all were classified into a new GBV-C group we provisionally designated GBV-C genotype 7. Lastly, three full-length GBV-C genomes were characterized; it provides us the ultimate information for defining a new genotype.

Of this study, the GBV-C genotype distribution pattern is vastly different from that previously reported in China where genotype 3 is predominant [23], [24]. This pattern looks also different from that observed in Southeast Asia where genotypes 4 and 6 are common [10]. Co-analysis of reference 5′UTR sequences showed that GBV-C genotype 7 was also found in Southeast Asia, albeit not common. The basal location of Southeast Asia sequences in phylogenetic tree implies that GBV-C genotype 7 may be indigenous in this region. Recently, an overland drug trafficking route may have exchanged viral strains with those in Southeast Asia. Through Yunnan as a center, this route has been proven to play a critical role in the transmission of HIV-1 and HCV infection from Southeast Asia to the other parts China [43], [44]. Mutually, this route may have also spread the infection with GBV-C between Yunnan and its neighboring countries. Up to now, GBV-C genotype 7 has not been found in other provinces marginal to Yunnan, such as Guangxi and Sichuan, where genotype 3 was the only discovered GBV-C strains major due to the lake of recent GBV-C investigation there. However, the spread of this novel GBV-C genotype through modern transmission route could be predicted.

In this study, GBV-C genotype 7 was designated by following the system Mueroff and his colleagues had recommended [10], [45]. However, based on the analysis of E2 region sequences and/or full-length genomes, five major GBV-C groups may be proposed. Four groups are represented by genotypes 1, 2, 3 and 5, respectively; while the fifth group is composed of genotypes 4, 6 and the newly designated GBV-C genotype 7. For more than a decade, GBV-C had been well classified into four genotypes: 1–4. This has been established with sequences having origins from exclusive geographic regions: genotype 1 from Africa, genotype 2 from Europe and America, genotype 3 from East Asia, and genotype 4 from Southeast Asia. Recently, genotype 5 has been proposed with sequences from Africa [45]. Genotype 6 has been separated from genotype 4 with sequences from Indonesia and Japan [10]. Using a similar approach, we also separated an Indonesian isolate, IndHD92, from a previous grouping into genotype 4 as a now grouping into genotype 7 (Figure 3). Genotypes 4, 6, and 7 can compose a larger genetic group (Figure 2), which geographically from Southeast Asia or its neighboring region such as Yunnan in the present study. These distribution patterns indicate their common origins. Unlike many other viruses, currently there is a lack of consented and quantified criteria set for GBV-C classification of nucleotide sequences. Even though such a standard is established, it may not always meet the increasing need of classifying new viral variants. For example, new variants are kept being identified for HCV. However, a previously quantified criterion failed to classify them consistently, which had caused a confusion of six or 11 HCV genotypes. For this reason, a consensus paper has been subsequently modified, which now uniformly classifies HCV isolates based on phylogenetic analyses of their genetic sequences, no matter how greater genetic distances may be obtained [46]. This wisdom should be also applied for GBV-C classification, we strongly recommend. Following this scheme, all GBV-C isolates would be classified into five major groups to assist for a simpler GBV-C nomenclature. On the other hand, virus genetic variation is a reflection of the continuous mutations accumulated over the past history of viral evolution. Spatial niches with variants of different evolutionary extents do exist. However, due to limited sampling and technical restriction, many of such variants have not been identified. These variants may present continuous genetic variations to fill the gaps between GBV-C genotypes 4, 6, and 7, we strongly believe. As more such variants are characterized, a simpler GBV-C nomenclature may be modified.

A benefit of GBV-C infection on disease progression of AIDS or HIV-1 replication has been documented [18]. However, the impact of GBV-C genotypes on the progression of HCV or HIV-1 related diseases remains to be investigated. Although a few reports have described that GBV-C genotype 2 (2a and 2b) and 5 may be in association with a better immunological response among patients co-infected with HIV-1 [12], [16], there is a still lack of evidence supporting that GBV-C genetic diversity affects the HIV-1 caused clinical presentation. The identification of GBV-C genotype 7 and its predominance among IDUs enhanced our curiosity: if there is a correlation between GBV-C genotypes and the presence of clinical AIDS markers, which include CD4^+^ cell count and HIV-1 viral load. In addition, there is a need of investigating the correlation between GBV-C genotypes and HIV-1 subtypes or circulating recombinant forms (CRFs). As cousins of *Flaviviridea*, GBV-C and HCV have similar genome organization. A potential association between the two viruses in the context of genetic diversity and evolution also need to be investigated.

## Materials and Methods

### Subjects and specimens

A total of 120 IDUs were recruited, from whom anti-coagulated blood samples were collected. These IDUs were retained in six drug detoxification centers in five prefectures of the Yunnan province, China, during 2005–2008. Among them, 30 were from Kunming, 46 from Honghe, 13 from Wenshan, 15 from Dehong, and 16 from Dali (Table S1). This study and the consent procedure were approved by the local ethical review committee at the Yunnan Center for Disease Control and the Kunming University of Science and Technology. Verbal consent was obtained from recruited IDUs. Written consent was not necessary because no personal information, which may impact the life and reputation of attender, was recorded. The personal information was not included in publication. Serum anti-HCV, anti-HIV-1, and anti-E2 of GBV-C were tested using enzyme immunoassays (ELISA; Kehua Company, Shanghai, China; GBV-C/HGV env kit, Roche Diagnostics, Sydney, Australia). Samples positive for anti-HIV-1 were further confirmed using Western blot assay (BioRad, Singapore).

### Detection of GBV-C 5′UTR and E2 region sequences

Viral RNA was extracted from 200 µl plasma using High Pure Viral RNA Kit (Roche Applied Science). This was followed by RT-nested-PCR using the primers listed in Table S2 that targeted the GBV-C 5′UTR and E2 region. The first round RT-PCR was performed with 4 pmol of each outer primer using the AMV RNA PCR Kit (TaKaRa, Ver. 3.0). The second round PCR was conducted with 4pmol of each inner primer using the Premix Taq Kit (TaKaRa, EXTaqTM Ver). The samples with visible bands of predicted size were considered positive for GBV-C and the DNA was recovered.

### Amplification of the full-length GBV-C genome

From three GBV-C RNA + samples, full-length GBV-C genomes were characterized. To amplify the GBV-C complete genome, 51 primers were designed based on the aligned reference sequences. Their sequences were listed in Table S2 and the strategy was shown in Figure S2, generating 13 overlapping fragments for each isolate.

### Sequencing and phylogenetic analysis

All the amplicons were directly sequenced, and sequence information was analyzed using the BLAST, CLUSTAL_W, BioEdit and Mega 4 software. In more detail, the obtained sequences were confirmed as GBV-C using the NCBI (National Center for Biotechnology Information) BLAST searching program (http://www.ncbi.nlm.nih.gov) and aligned with reference sequences using CLUSTAL_W program. Further adjustments to the alignments were manually made using BioEdit program. Bootstrap resampling was performed with 1000 neighbour-joining replicates. Phylogenetic analysis of 5′UTR, E2 region, and full-length sequences was conducted using Mega 4 software, which employed Jukes-Cantor distances of neighbour-joining tree.

### Nucleotide sequence accession numbers

The sequences reported in this paper have been deposited in GenBank with the following accession numbers: HQ331171 to HQ331235.

## Supporting Information

Figure S1 **GBV-C, HCV and HIV-1 mono, dual or triple infection rates.** The symbol ** indicates the rate of GBV-C/HIV-1/HCV triple infection was significantly higher than that of GBV-C/HIV-1 and GBV-C/HCV dual infection.
(TIF)

Figure S2 **Strategies used to amplify the KY117, DL185 and DH028 complete genomes.** The bar at top represents the genomic organization of GBV-C and shows the seven protein encoding regions of various lengths, flanked by the 5′ and 3′ UTRs. Nucleotide numbering is according to the U36380 genome. Arrows represent the overlapping fragments amplified for the three GBV-C isolates.
(TIF)

Table S1 **The infection rates of GBV-C/HCV/HIV-1 among recruited IDUs in Yunnan province.**
(DOC)

Table S2 **Primers used for amplification and sequencing of the GBV-C genome.**
(DOC)
