# Supplementary figures and images for: A Novel Genotype of GB Virus C: Its Identification and Predominance among Injecting Drug Users in Yunnan, China
Source: PLoS One. 2011 Oct 6;6(10):e21151. doi: 10.1371/journal.pone.0021151 (PMC3188531; doi:10.1371/journal.pone.0021151)

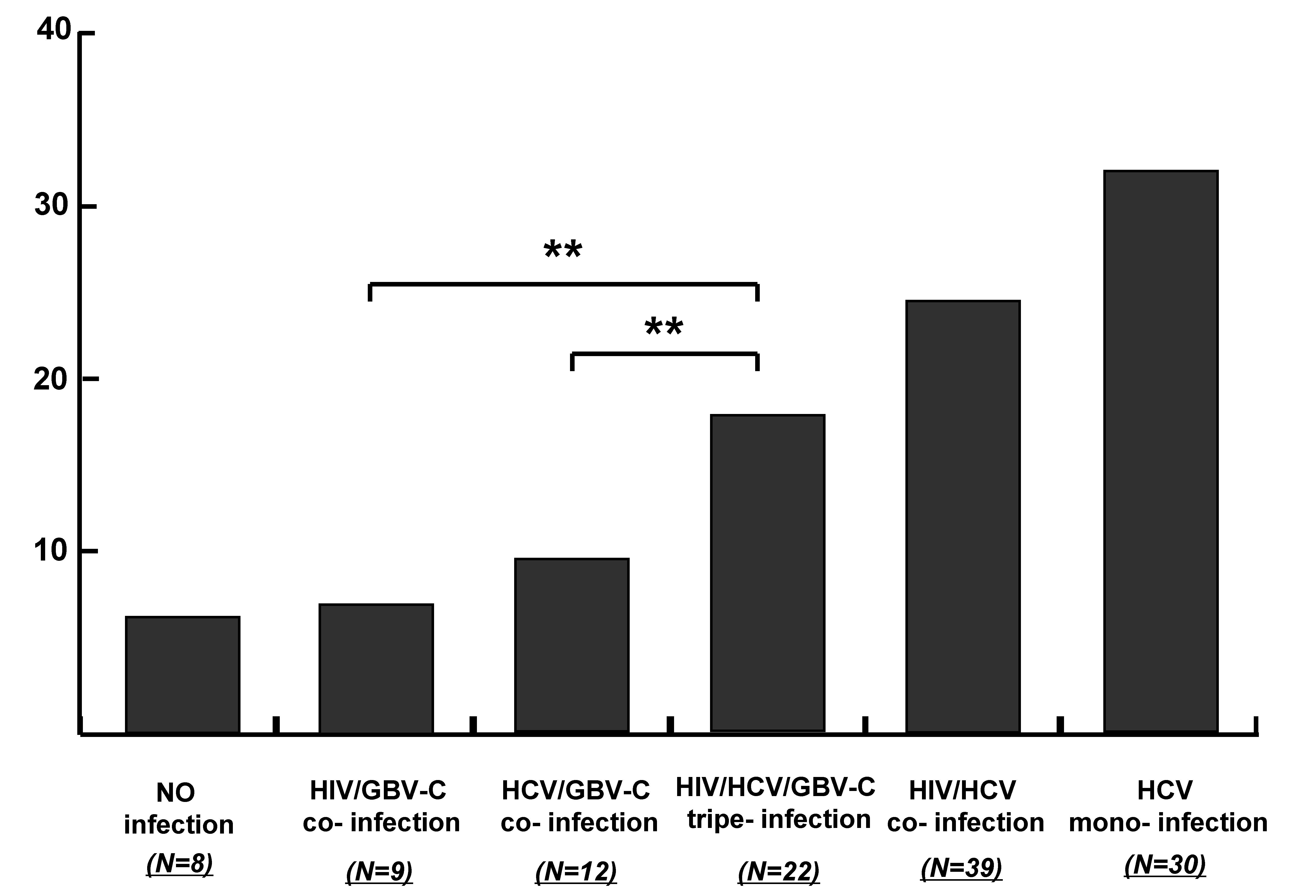

Supplement: Figure S1 — GBV-C, HCV and HIV-1 mono, dual or triple infection rates. The symbol ** indicates the rate of GBV-C/HIV-1/HCV triple infection was significantly higher than that of GBV-C/HIV-1 and GBV-C/HCV dual infection. (TIF) [file pone.0021151.s001.tif]

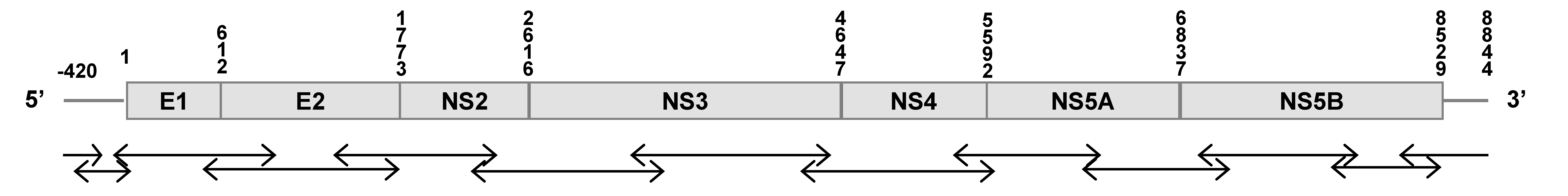

Supplement: Figure S2 — Strategies used to amplify the KY117, DL185 and DH028 complete genomes. The bar at top represents the genomic organization of GBV-C and shows the seven protein encoding regions of various lengths, flanked by the 5′ and 3′ UTRs. Nucleotide numbering is according to the U36380 genome. Arrows represent the overlapping fragments amplified for the three GBV-C isolates. (TIF) [file pone.0021151.s002.tif]
