# Supplementary material for: A Novel Genotype of GB Virus C: Its Identification and Predominance among Injecting Drug Users in Yunnan, China
Source: PLoS One. 2011 Oct 6;6(10):e21151. doi: 10.1371/journal.pone.0021151 (PMC3188531; doi:10.1371/journal.pone.0021151)
Supplement: Table S1 — The infection rates of GBV-C/HCV/HIV-1 among recruited IDUs in Yunnan province. (DOC) [file pone.0021151.s003.doc]

**Table S1. The infection rate of GBV-C/HCV/HIV-1 among IDUs**

| Geographic area | Dehong  (N=15) | Dali  (N=16) | Kunming  (N=30) | Honghe  (N=46) | Wenshan  (N=13) | Total(%)  (N=120) |
| --- | --- | --- | --- | --- | --- | --- |
| Age | 30±4.38 | 33±10.23 | 31±9.81 | 34±5.82 | 27±4.13 | 33±6.78 |
| Male/Female | 11:4 | 14:2 | 27:3 | 38:8 | 12:1 | 102:18 |
| Anti-GBV-C E2^+^ | 7 | 4 | 7 | 12 | 1 | 31/(25.8) |
| GBV-C RNA^+^ | 8 | 1 | 14 | 18 | 2 | 43/(35.8) |
| Anti-E2^+^/GBV-C RNA^-^ | 7 | 4 | 6 | 11 | 1 | 29/(24.2) |
| Anti-E2^+^/GBV-C RNA^+^ |  |  | 1 | 1 |  | 2/(1.7) |
| Anti-HCV^+^ | 15 | 16 | 27 | 32 | 13 | 103/(85.8) |
| Anti-HIV^+^ | 14 | 8 | 16 | 20 | 12 | 70/(58.3) |
