# Supplementary material for: A Novel Genotype of GB Virus C: Its Identification and Predominance among Injecting Drug Users in Yunnan, China
Source: PLoS One. 2011 Oct 6;6(10):e21151. doi: 10.1371/journal.pone.0021151 (PMC3188531; doi:10.1371/journal.pone.0021151)
Supplement: Table S2 — Primers used for amplification and sequencing of the GBV-C genome. (DOC) [file pone.0021151.s004.doc]

**Table S2. List of the nested PCR primers used in this study**

| No. ^a^ | Name ^b^ | | Sequences (5’-3’) | Position ^c^ | Sizes ^d^ |
| --- | --- | --- | --- | --- | --- |
| 1 | Outer primer | G01F1 | CTGGGTGCAAGCCCCAGAAACCG | -518~-496 | 257 |
|  |  | G01R1 | CCACTGGTCCTTGTCAACTCG | -152~-172R |  |
|  | Inner primer | G01F2 | CCAAAAGGTGGTGGATGGGTGAT | -447~-425 |  |
|  |  | G01R2 | CAAGAGAGACATTGAAGGGCGAC | -191~-213R |  |
|  |  |  |  |  |  |
| 2 | Outer primer | G02F1 | GGTTGGTAGGTCGTAAATCCCG | -419~-398 | 378 |
|  |  | G02R1 | AATGCCACCCGCCCTCA | -18~-34R |  |
|  | Inner primer | G02F2 | GTAGGTCGTAAATCCCGGTCA | -414~-394 |  |
|  |  | G02R2 | CGAAGGTTCTTGGGCTACC | -37~-55R |  |
|  |  |  |  |  |  |
| 3 | Outer primer | G03F1 | TCCACGTCGCCCTTCAATGT | -218~-199 | 1061 |
|  |  | G03R1 | GGAAGCAAACCAAGACACGGATC | 924~902R |  |
|  | Inner primer | G03F2 | CGTCGCCCTTCAATGTCTCTCTT | -214~-192 |  |
|  |  | G03R2 | CCACTGATTTTGTCCGTGGCTC | 846~825R |  |
|  |  |  |  |  |  |
| 4 | Outer primer | G04F1 | GCGTCCTCACTGTGGGYGTTG | 326~346 | 1300 |
|  |  | G04R1 | GGATCATACTSACGAMGGGWAGG | 1837~1857R |  |
|  | Inner primer | G04F2 | TGGGAGAGTGAGTTTTGGAGATGG | 409~432 |  |
|  |  | G04R2 | GGAGRAGTATRAGCGGGACCAAC | 1708~1686R |  |
|  |  |  |  |  |  |
| 5 | Outer primer | G05F1 | GTCCTACACCATGACCAAGATCC | 1287~1309 | 1063 |
|  |  | G05R1 | CGACAYTCCGTCCTAGTGAAWG | 2543~2522R |  |
|  | Inner primer | G05F2 | TGAAATGTCCCACYCCTGCC | 1328~1347 |  |
|  |  | G05R2 | TCRCCAGCCATCACACARC | 2390~2372R |  |
|  |  |  |  |  |  |
| 6 | Outer primer | G06F1 | CGTGGTGYAARGGRTAYCAGG | 2129~2149 | 1273 |
|  |  | G06R1 | GTCTCAATGATGGANGGGTGCTG | 3698~3678R |  |
|  | Inner primer | G06F2 | GAYGCYGTRATGHTGGTGGTG | 2245~2265 |  |
|  |  | G0R2 | GCCTAGGGTTGGCAAGRAAC | 3517~3498R |  |
|  |  |  |  |  |  |
| 7 | Outer primer | G07F1 | GAATGCTSGTGTCMGTGCTTCAYTC | 3131~3155 | 1302 |
|  |  | G07R1 | CATCCCARTCTGTHACCACCAC | 4657~4636R |  |
|  | Inner primer | G07F2 | CATGGGSCACAARGTCCTBAT | 3330~3350 |  |
|  |  | G07R2 | GASCCRGTGTAWGACGCRTAGATCAT | 4631~4606R |  |
|  |  |  |  |  |  |
| 8 | Outer primer | G08F1 | TGGGTGTTCAGCGGACSATGT | 4361~4381 | 1262 |
|  |  | G08R1 | AGRCAGCGRCTYTCCACATG | 5759~5740R |  |
|  | Inner primer | G08F2 | CCRAATCCTGTCCCRYTACTGC | 4441~4462 |  |
|  |  | G08R2 | CARTGCCACAAAGGDAGYGAC | 5702~5682R |  |
|  |  |  |  |  |  |
| 9 | Outer primer | G09F1 | TCAGCWAACAACTCWGGCACT | 5401-5421 | 963 |
|  |  | G09R1 | TCTGAGCTGCTCTCCGTAACC | 6449~6429R |  |
|  | Inner primer | G09F2 | TGYATYCCRGACAGYTAYTTCCAAC | 5467~5491 |  |
|  |  | G09R2 | CGAGATAAGTGCAGGCGATGG | 6429~6409R |  |
|  |  |  |  |  |  |
| 10 | Outer primer | G10F1 | TCCARGCBATHGAGAATGCTG | 6278~6298 | 965 |
|  |  | G10R1 | TGRTTKGGGGTGTACTGGAAGGC | 7415~7393R |  |
|  | Inner primer | G10F2 | TGTCATCATGGAGGAYTGYAGTACAC | 6324~6349 |  |
|  |  | G10R2 | CCTTCTCCTCCTTWCGGTCT | 7288~7269R |  |
|  |  |  |  |  |  |
| 11 | Outer primer | G11F1 | GAGGAGGCAATAAGGACTGTYAG | 7093~7115 | 1047 |
|  |  | G11R1 | CCACGATGATGTTRGGCAGTT | 8179~8159R |  |
|  | Inner primer | G11F2 | CAATAAGGACTGTHAGGCCRC | 7100~7120 |  |
|  |  | G11R2 | ACTTGTAGTARTTRCCATGHACCTG | 8146~8122R |  |
|  |  |  |  |  |  |
| 12 | Outer primer | G12F1 | CATGTCSAGYGAGTACAGTGAYCC | 7968~7991 | 542 |
|  |  | G12R1 | GTTAGTAACCACCATGCCTCCCAAG | 8662~8638R |  |
|  | Inner primer | G12F2 | GAGTACAGYGAYCCWATGGCT | 7978~7998 |  |
|  |  | G12R2 | GCYACGATGAGCAGGGYTAAG | 8519~8499R |  |
|  |  |  |  |  |  |
| 13 | Outer primer | G13F1 | GYCAGGTKCATGGYAAYTACTACAAGT | 8120~8146 | 642 |
|  |  | G13R1 | AAAAAAAAAGTAGAACCCGGCCTTTGG | 8844~8826R |  |
|  | Inner primer | G13F2 | CGCAGACACAACYAAAACMAAAATG | 8211~8235 |  |

^a^Genomic fragment amplified. ^b^Primers used for PCR amplification. ^c^Nucleotide positions with reference to the numbering of the U36380 genome. ^d^Expected sizes of the amplicon in bp.
